# Prognostic evaluation and immune infiltration analysis of five bioinformatic selected genes in hepatocellular carcinoma

**DOI:** 10.1111/jcmm.17035

**Published:** 2021-11-02

**Authors:** Enjiang Lai, Yang Tai, Jingsun Jiang, Chong Zhao, Yang Xiao, Xin Quan, Hao Wu, Jinhang Gao

**Affiliations:** ^1^ Lab of Gastroenterology and Hepatology West China Hospital Sichuan University Chengdu China; ^2^ Department of Gastroenterology West China Hospital Sichuan University Chengdu China

**Keywords:** bioinformatics, cell proliferation, hepatocellular carcinoma, immune infiltration, prognosis

## Abstract

Despite the development in hepatocellular carcinoma (HCC) treatment in recent years, the therapeutic outcome of HCC remains unfavourable. This study examines the prognosis of HCC from a genetic level using clinical databases and single‐cell data to identify genes with a high prognostic value. Three up‐regulated genes (*UBE2S*, *PTTG1*, and *CDC20*) and two down‐regulated genes (*SOCS2* and *DNASE1L3*) in HCC tissues were identified. Various analyses confirmed its correlation with tumour stage (*p *< 0.01) and patient survival time (log‐rank *p *< 0.001). Immune analysis, single‐cell analysis, and gene set enrichment analysis (GSEA) were employed to provide insight on how they affect cancer progression, and we observed a close relation between these genes and tumour immune infiltration. Eventually, we constructed a risk score system that risk score = (0.0465) × *UBE2S* + (0.1851) × *CDC20* + (−0.0461) × *DNASE1L3* + (−0.2279) × *SOCS2* (5‐year area under curve = 0.706). The risk score system may serve as an effective novel prognostic system for HCC patients. This study might provide novel ideas for prognostic or therapeutic biomarkers for HCC.

## 1 INTRODUCTION

Primary liver cancer is the second leading cause of cancer‐related death with an increasing global incidence.^1^ Hepatocellular carcinoma (HCC) is the most common type of primary hepatic malignancy that accounts for approximately 70%–80% of all primary liver cancers.^1^, ^2^ Although early HCC can be treated by liver resection or transplantation, such options may not be available for cases with an advanced stage at the time of diagnosis.^3^, ^4^, ^5^ Even after curative‐intent surgical resection, the 5‐year survival rate of HCC patients remains poor due to the high recurrence rate.^6^ Therefore, accurate estimation of the prognosis is crucial for clinical decision‐making and personalized treatment. Traditional prognostic prediction for HCC mainly relies on pathological grade and tumour node metastasis (TNM) stage, which is insufficient to predict the outcome of patients. Thus, it is urgent to explore more accurate biomarkers for the early prediction and prognosis evaluation of HCC.

Recently, the development of high‐throughput genetic technology has revolutionized the landscape of oncology research, allowing us to study tumour biology at the molecular level.^7^ Genetic research has led to substantial advancements in the diagnosis and treatment of various cancers, such as breast cancer, prostate cancer, and colon cancer.^8^, ^9^, ^10^, ^11^ Identification of key genes in tumours not only reveals the mechanism of tumorigenesis and cancer progression, but also provides therapeutic and prognostic targets for precision and personalized medicine. Previous studies of HCC genetics have mainly focused on the prognostic value of known oncogenic or/and tumour‐suppressor genes.^12^, ^13^, ^14^ Some studies have approached this topic by analysing the differentially expressed genes (DEGs) in HCC and normal tissues, but few of them combined the findings with single‐cell analysis of the genes to explain the mechanism behind their effect on tumour progression. Increasing evidence indicates that the tumour immune microenvironment plays a pivotal role in the development and prognosis of cancers.^15^ However, the genetics behind this process in HCC remains to be fully discovered.

In this study, we explored the prognosis‐related DEGs in HCC based on gene expression profiles from multiple databases and analysed their correlation with clinicopathological characteristics, immune infiltration, and patient survival. Eventually, we constructed a prognostic model using these genes to predict clinical outcomes in patients with HCC.

## 2 MATERIALS AND METHODS

### 2.1 Data acquisition and processing

Gene Expression Omnibus (GEO, https://www.ncbi.nlm.nih.gov/geo/) is an international repository that archives microarray, next‐generation sequencing, and other forms of high‐throughput functional genomics data. The transcriptome profiles and clinical information of HCC patients were obtained from GEO databases, including 115 cases from GSE76427, 24 cases from GSE101685, and 183 cases from GSE112790.^16^, ^17^, ^18^ Differential expression analysis was performed using the “DESeq2” R package by the standard of adjusted *p* value < 0.01 and |log2 (fold change)| >1. A Venn diagram was used to generate the overlapped DEGs.

### 2.2 Gene Expression Profiling Interactive Analysis (GEPIA)

Gene Expression Profiling Interactive Analysis (http://gepia.cancer‐pku.cn/index.html) is a web server for cancer and normal gene expression profiling and interactive analyses based on The Cancer Genome Atlas (TCGA) and the Genotype‐Tissue Expression (GTEx) data integration.^19^ In this study, survival‐related genes obtained from GEPIA were used to identify the target genes that overlapped with DEGs from GEO databases. Additionally, gene expression profiles according to cancer types or pathological stages and survival analysis were also performed by GEPIA or GEPIA2 (http://gepia2.cancer‐pku.cn/#index). A Sankey diagram was constructed to integrate gene expression, clinicopathological characteristics, and prognosis using the “ggalluval” R package.

### 2.3 Survival analysis by Kaplan–Meier Plotter

The prognostic value of the target genes was further validated by an open‐access bioinformatic tool Kaplan–Meier Plotter (http://kmplot.com/analysis/),^20^ in which 364 HCC cases were classified into the high‐ or low‐expression group according to various quantile expressions of the proposed biomarker. Then, they were compared by a Kaplan–Meier survival plot, and the hazard ratio with 95% confidence intervals and log‐rank *p* value were calculated. A *p* value < 0.05 was considered statistically significant.

### 2.4 Gene interaction analysis by STRING and GeneMANIA

The Spearman's correlation analysis between the target genes was plotted as a heatmap using the “pheatmap” R package. Protein–protein interaction (PPI) analysis of them was further performed using STRING (http://string‐db.org/)^21^ and GeneMANIA (http://genemania.org/)^22^ online tools that predict functional interaction networks based on multiple databases.

### 2.5 Gene co‐expression and pathway enrichment analysis by LinkedOmics

LinkedOmics (http://www.linkedomics.org/) is a publicly available portal that provides a visual platform for biologists and clinicians to access, analyse, and compare multiomics data from all 32 TCGA cancer types.^23^ Gene co‐expression analysis with the target genes in HCC was performed using Pearson's correlation coefficient, presenting in scatter plots and heatmaps. Reactome pathway enrichment of the co‐expressed genes was then generated from the LinkedOmics database. Additionally, Gene Set Enrichment Analysis (GSEA) was performed in GenePattern using curated gene sets from the Reactome database.

### 2.6 Mutation analysis by cBioPortal

The cBioPortal for Cancer Genomics (http://www.cbioportal.org/) is an open‐access, open‐source resource for interactive exploration of multidimensional cancer genomics datasets.^24^ It integrates data from 126 tumour genome studies, including large tumour research projects such as TCGA and International Cancer Genome Consortium (ICGC). A color‐coded map of genetic alterations in the target genes of HCC patients was constructed using OncoPrinter through cBioPortal TCGA datasets.

### 2.7 Immune infiltration analysis by TISIDB and TIMER

TISIDB (http://cis.hku.hk/TISIDB) is a web portal for tumour and immune system interaction, which integrates multiple types of data resources in oncoimmunology.^25^ TIMER (http://timer.cistrome.org/) is a comprehensive resource for immune infiltration analysis across diverse cancer types.^26^ In this study, the correlation of gene expression with immune features (immunomodulators, chemokines, and chemokine receptors) and immune cell infiltration levels was evaluated with TISIDB and TIMER, respectively. Moreover, partial Spearman's correlation analysis with the quanTIseq method was also performed for each immune cell subtype to reveal the relationship between infiltrates estimation value and gene expression in HCC samples.

### 2.8 Single‐cell RNA‐sequencing analysis

Human Liver Browser (http://itzkovitzwebapps.weizmann.ac.il/webapps/home/session.html?app=HumanLiverBrowser) and Single‐cell Atlas in Liver Cancer (scAtlasLC, https://scatlaslc.ccr.cancer.gov/) are public databases of single‐cell transcriptomic profiles for HCC.^27^, ^28^ The expression of the target genes in malignant and non‐malignant cells in HCC was analysed by Human Liver Browser and scAtlasLC.

### 2.9 Construction of prognostic signature and internal validation

Univariate and multivariate Cox regression analyses were performed to identify the target genes related to prognosis in the TCGA cohort. Then, the least absolute shrinkage and selection operator (LASSO) regression model with tenfold cross‐validation was performed to identify the most significant survival‐related genes. Stepwise multivariate Cox regression analysis was applied to further establish the prognostic signature in HCC. The risk score was calculated by the following formula: risk score = expression of gene 1 × coefficient 1 + expression of gene 2 × coefficient 2 + … expression of gene n × coefficient n. To validate the predictive ability, all HCC patients were allocated into high‐ or low‐risk groups according to the median value of the risk score. Kaplan–Meier curve analysis and log‐rank test were performed to compare the overall survival difference between the two groups using the “Survival” R package. In addition, the receiver operating characteristic (ROC) model was also utilized to evaluate the predictive power of this prognostic signature.

## 3 RESULTS

### 3.1 Identification of prognosis‐related DEGs in HCC

**FIGURE 1:**
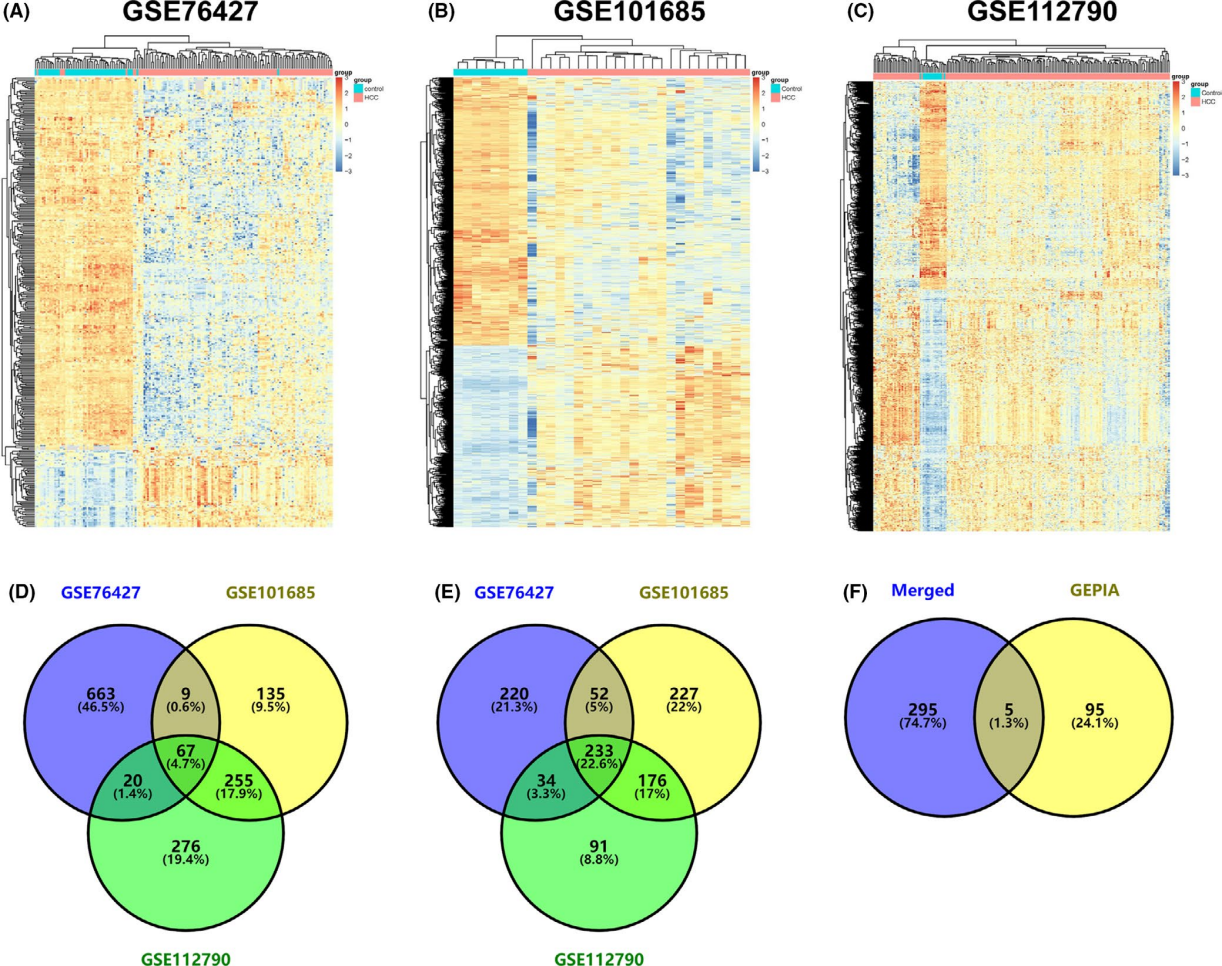
Identification of prognosis‐related DEGs in HCC. (A–C) Heatmaps of the DEGs associated with HCC in GSE76427 (A), GSE101685 (B), and GSE112790 (C). (D–E) The overlapped genes of the up‐regulated DEGs (D) and down‐regulated DEGs (E) from the GEO cohorts. (F) The five identified prognosis‐related DEGs from the GEO and GEPIA databases

To identify the candidate genes related to HCC prognosis, the GEO and GEPIA databases were used to screen for DEGs associated with HCC (Figure 1). After taking intersections from different GEO cohorts (GSE76427, GSE101685, and GSE112790, Figure 1A–C), a total of 300 DEGs in HCC samples were identified, with 67 genes up‐regulated (Figure 1D) and 233 genes down‐regulated (Figure 1E). Then, the top 100 most significant survival genes were generated from the GEPIA database (Figure 1F). Finally, the overlapped genes with DEGs from GEO, including ubiquitin‐conjugating enzyme E2S (*UBE2S*), pituitary tumour‐transforming gene 1 (*PTTG1*), cell division cycle 20 (*CDC20*), suppressor of cytokine signalling 2 (*SOCS2*), and deoxyribonuclease 1 like 3 (*DNASE1L3*), were considered as prognosis‐related DEGs (target genes) and were subjected to subsequent analyses.

### 3.2 Transcriptional expression of the target genes in HCC

**FIGURE 2:**
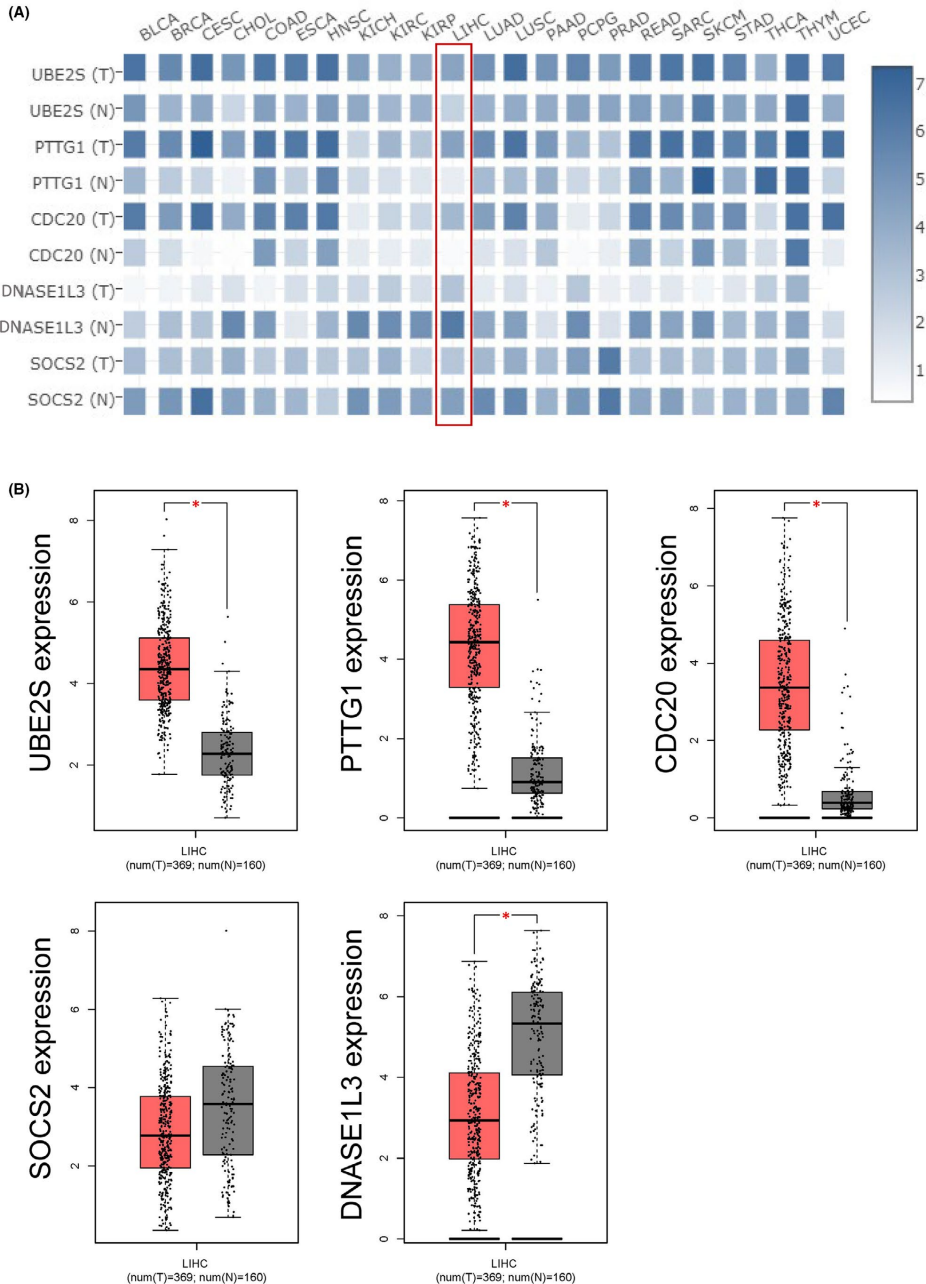
Transcriptional expression of the target genes in HCC. (A) Transcription levels of the five target genes in human cancers (GEPIA2). (B) Transcription levels of the five target genes in HCC (GEPIA). ^*^adj. *p* < 0.05

The transcriptional expression levels of the target genes in human cancers have been determined using the GEPIA database (Figure 2). As shown in Figure 2A, the expression levels of *UBE2S*, *PTTG1*, and *CDC20* were significantly higher in most cancer tissues than in normal tissues. The increased expression of *UBE2S*, *PTTG1*, and *CDC20* in HCC was also observed compared with that in normal liver (*p* < 0.05, Figure 2A,B). Conversely, *SOCS2* and *DNASE1L3* were down‐regulated in most cancer tissues compared to normal tissues (Figure 2A). The decreased expression of *DNASE1L3* was verified in HCC (*p* < 0.05, Figure 2A,B), while *SOCS2* was not significantly reduced in HCC tissues compared to normal livers (*p* > 0.05, Figure 2B).

### 3.3 Correlation of the target genes with clinicopathological characteristics and patient survival in HCC

**FIGURE 3:**
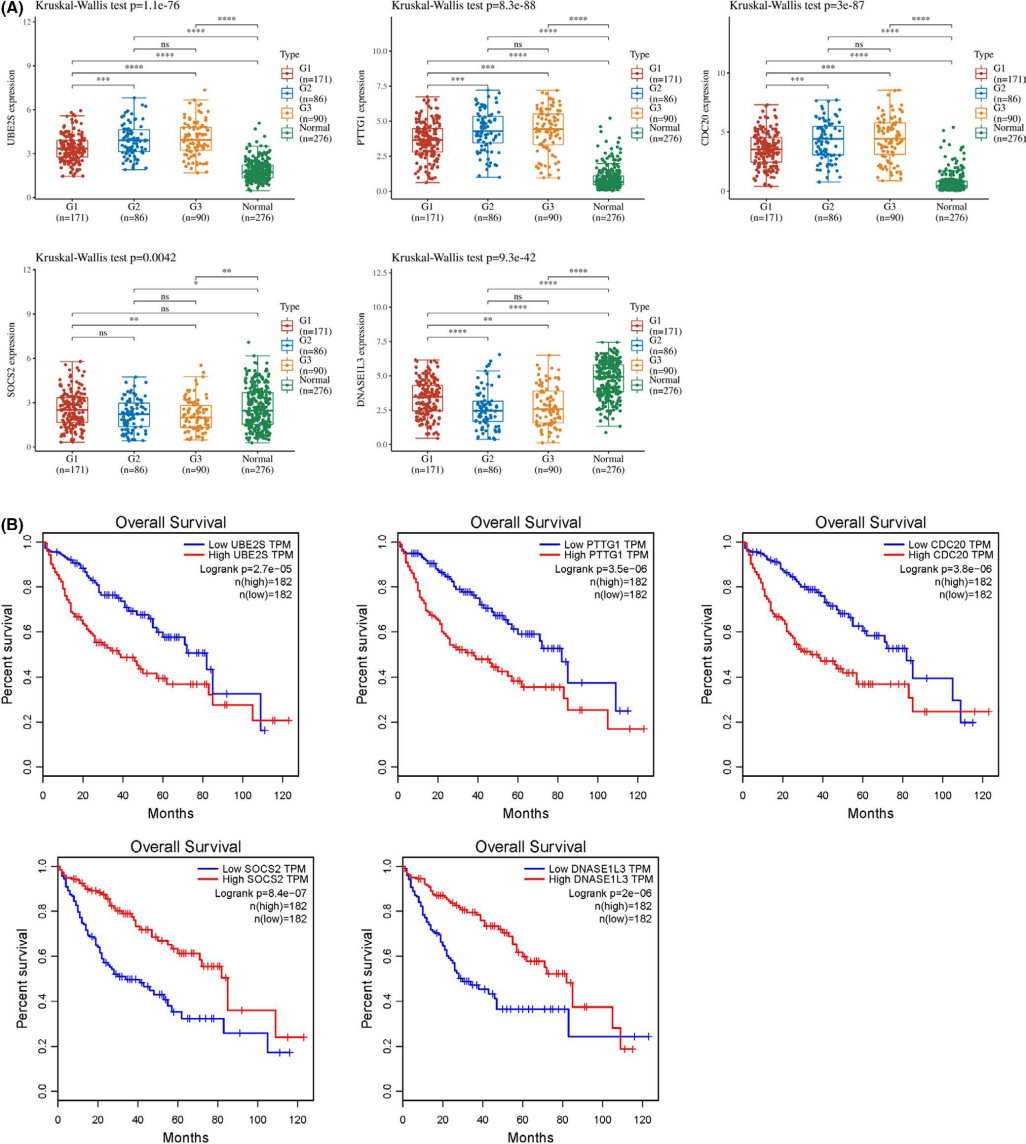
Correlation of the target genes with pathological stage and patient survival in HCC. (A) Correlation between the target genes expression and pathological stage in HCC (TCGA). (B) Prognostic value of the target genes in HCC (GEPIA). ^****^ *p* < 0.0001, ^***^ *p* < 0.001, ^**^ *p* < 0.01, ^*^ *p* < 0.05, ns, not significant

The TCGA database was used to evaluate the relationship between the target genes and the pathological stage of HCC patients. Kruskal–Wallis test showed that the expression levels of the five target genes (*UBE2S*, *PTTG1*, *CDC2*, *SOCS2*, and *DNASE1L3*) were significantly correlated with the pathological stage of HCC (*p* < 0.05, Figure 3A). Next, we used the GEPIA database and Kaplan–Meier plotter to further determine the prognostic values of the target genes in HCC patients. The survival curves revealed that higher levels of *UBE2S*, *PTTG1*, and *CDC20* expression predicted a poor prognosis, while higher expression of *SOCS2* and *DNASE1L3* predicted a better prognosis (*p* < 0.05, Figures 3B and S1). Additionally, Sankey diagrams using TCGA data were generated to better visualize the correlation between gene expression, clinicopathological characteristics, and prognosis in patients with HCC (Figure S2). It is clearly observed in the chart that patients with high levels of *UBE2S*, *PTTG1*, and *CDC20* were more likely to have a higher pathological stage and worse prognosis, while those with high levels of *SOCS2* and *DNASE1L3* were more likely to have the opposite tendency.

### 3.4 Gene interaction, co‐expression, and pathway enrichment of the target genes in HCC

**FIGURE 4:**
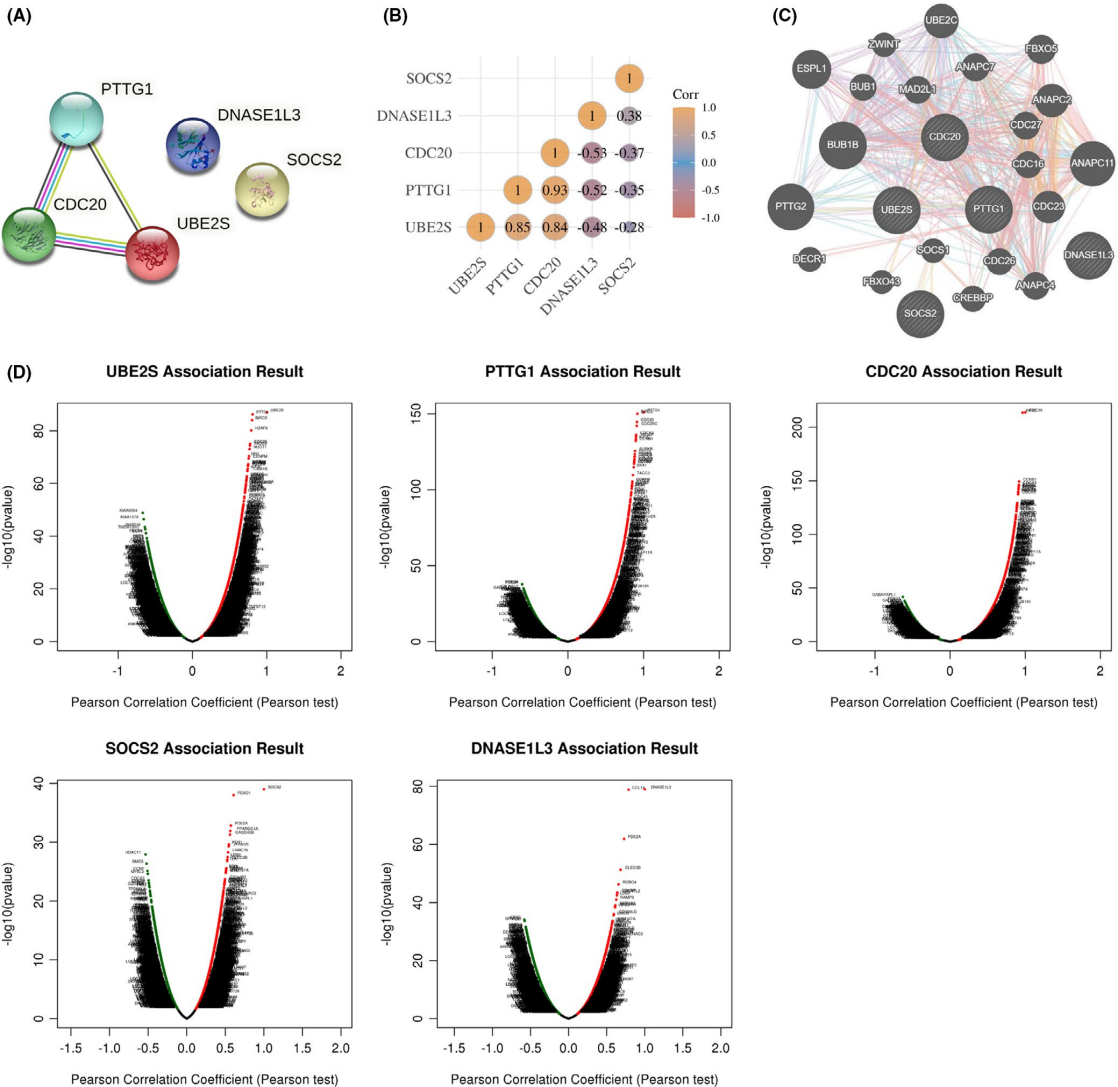
Gene interaction and co‐expression of the target genes in HCC. (A) Protein–protein interaction network of the five target genes (STRING). (B) Heatmap from the gene‐to‐gene Spearman's correlation for the five target genes (TCGA). (C) Gene interaction network of the five target genes and their related genes (GeneMANIA). (D) The related genes co‐expressed with the five target genes (LinkedOmics)

The correlation and interaction of the target genes were evaluated using the TCGA, STRING, and GeneMANIA databases (Figure 4A–C). When the target genes were mapped into the STRING database for PPI network analysis, the interactions between *UBE2S*, *PTTG1*, and *CDC20* were observed, while *SOCS2* and *DNASE1L3* did not interact with others (Figure 4A). A heatmap from the gene‐to‐gene correlation for the five target genes was then plotted according to Spearman's correlation analysis (Figure 4B). Furthermore, GeneMANIA network analysis revealed that *UBE2S*, *PTTG1*, *CDC20*, and *SOCS2* could physically interact and co‐express with each other (Figure 4C).

Next, LinkedOmics was employed to identify the related genes co‐expressed with the target genes and their biological functions. As shown in Figure 4D, the expression of *UBE2S*, *PTTG1*, and *CDC20* was positively correlated with each other. All of the five target genes have close associations with genes regulating cell cycle (*CDC23*, *ANAPC11*, and *ANAPC4*) and cell mitosis (*BUB1B*, *FBXO5*, *MAD2L1*, and *ESPL1*), which was consistent with pathway enrichment results (Figures S3–S7). Moreover, immune‐related pathways, such as complement cascade and its regulation, were also significantly enriched in the associated genes (Figures S3–S7).

The genetic alterations of the target genes in HCC patients were determined using the cBioPortal online tool. The results indicated that the target genes were low‐frequency mutated genes with the altered rate varying from 0.4% to 1.1% in the queried HCC samples (Figure S8).

### 3.5 Role of the target genes in HCC immune infiltration

**FIGURE 5:**
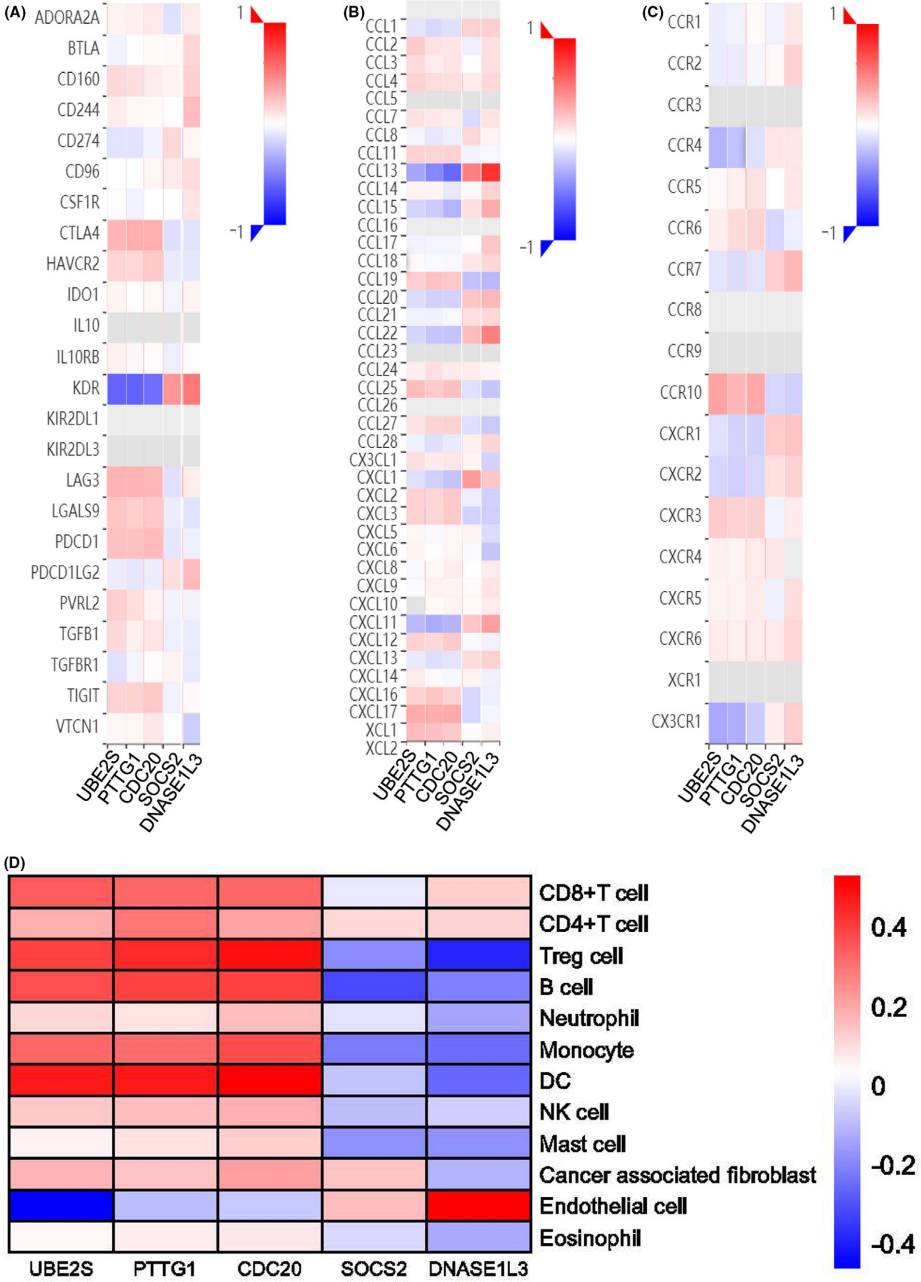
Immune infiltration analysis of the target genes in HCC. (A–C) Heatmaps of the correlation between the five target genes and immunomodulators (A), chemokines (B), and chemokine receptors (C) in HCC (TISIDM). (D) Heatmaps of the correlation between the five target genes and immune cells in HCC (TIMER)

As the tumour immune microenvironment plays a pivotal role in the tumorigenesis and progression of cancers, the TISIDB and TIMER databases were used to explore the impact of the target genes on immune features and immune cell infiltration in HCC. Firstly, the expression of the target genes was significantly correlated with immunomodulators (Figure 5A), chemokines (Figure 5B), and chemokine receptors (Figure 5C). Next, *UBE2S*, *PTTG1*, and *CDC20* were positively correlated with immune cells in HCC, while *SOCS2* and *DNASE1L3* were negatively correlated with immune cells in HCC (Figure 5D). In addition, the most positive correlation of *UBE2S*, *PTTG1*, and *CDC20*, and the most negative correlation of *SOCS2* and *DNASE1L3* were observed in regulatory T cells (Treg cells), B cells, monocytes, and dendritic cells (DCs). To further explore the relationship between gene expression and individual immune cells, partial Spearman's correlation analysis was then performed using the quanTIseq method (Figure S9). The results showed a significant positive correlation between *UBE2S*, *PTTG1*, and *CDC20* expression and infiltration levels of CD4^+^ T cells, CD8^+^ T cells, Treg cells, B cells, monocytes, and natural killer (NK) cells. In comparison, *SOCS2* showed a significant negative correlation with infiltration levels of DCs and NK cells. Additionally, *DNASE1L3* was found to have a significant negative correlation with infiltration levels of neutrophils, monocytes, and NK cells. These findings indicated a close relationship between the target genes and immune infiltration in HCC.

### 3.6 Single‐cell analysis of the target genes in HCC

**FIGURE 6:**
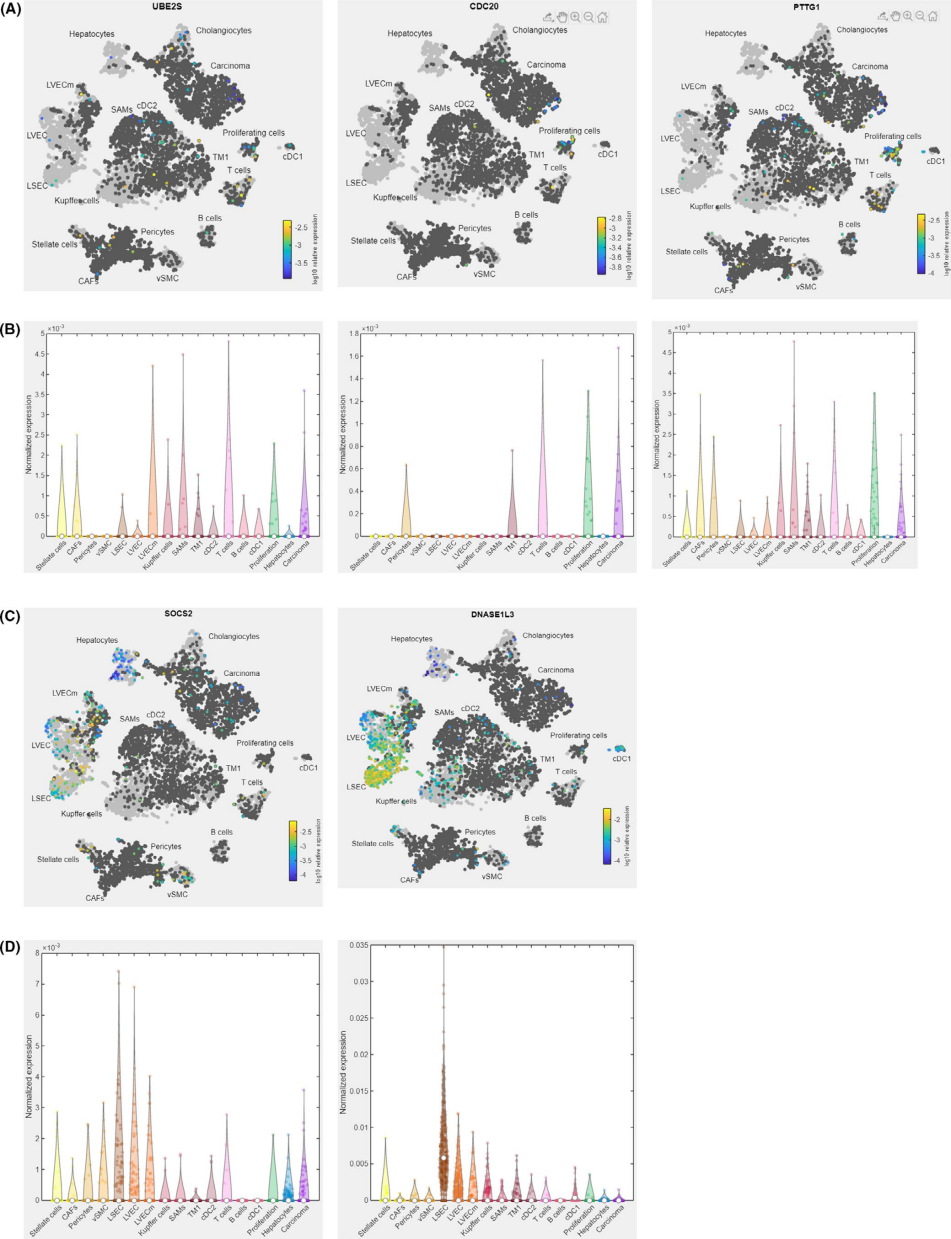
Single‐cell analysis of the target genes in HCC. (A, B) t‐SNE analysis (A) and expression levels (B) of *UBE2S*, *CDC20*, and *PTTG1* in liver cells (Human Liver Browser). (C,D) t‐SNE analysis (C) and expression levels (D) of *SOCS2* and *DNASE1L3* in liver cells (Human Liver Browser). CAFs, cancer‐associated fibroblasts; cDC, conventional dendritic cell; LSEC, liver sinusoidal endothelial cell; LVEC, lymphatic vascular endothelial cell; LVECm, malignant lymphatic vascular endothelial cell; SAMs, scar‐associated macrophages; TM, tissue monocytes; vSMC, vascular smooth muscle cell

To further explore the expression of the target genes in specific liver cells within HCC, we ran a combined t‐distributed stochastic neighbour embedding (t‐SNE) analysis from the Human Liver Browser (Figure 6) and scAtlasLC (Figure S10) datasets. It is revealed that *UBE2S* was mainly expressed in T cells, scar‐associated macrophages (SAMs), malignant lymphatic vascular endothelial (LVECm) cells, and carcinoma cells (Figure 6A,B). While *CDC20* was highly expressed in carcinoma cells, proliferation cells, T cells, tissue monocytes 1(TM1), and pericytes (Figure 6A,B). The expression of *PTTG1* was more evenly distributed in immune cells, while carcinoma cells showed moderately high levels of *PTTG1* (Figure 6A,B). *SOCS2* was mainly expressed in liver sinusoidal endothelial cells (LSECs) and lymphatic vascular endothelial cells (LVECs), while moderately expressed in carcinoma cells (Figure 6C,D). The expression level of *DNASE1L3* was exceptionally high in LSECs, while it was low in carcinoma cells (Figure 6C,D). The above results indicated that the target genes were differentially expressed in various immune cell types.

### 3.7 Construction of target genes‐based prognostic signature and internal validation in HCC

**FIGURE 7:**
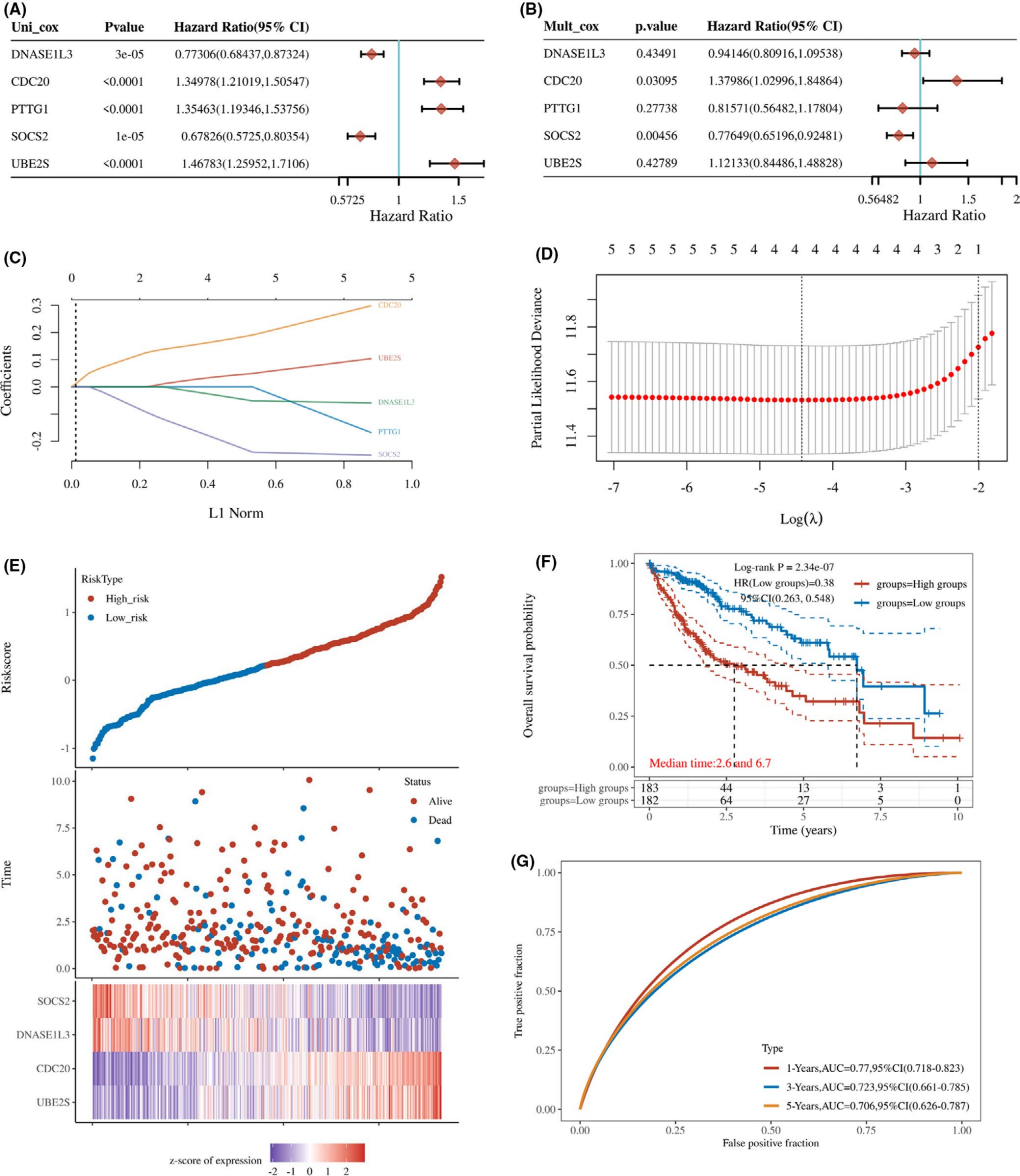
Construction of target genes‐based prognostic signature and internal validation in HCC. (A, B) Univariate (A) and multivariate (B) Cox regression analyses of the five target genes and clinical prognosis in HCC (TCGA). (C) Selection of the optimal parameter (lambda) in the least absolute shrinkage and selection operator (LASSO) model. (D) LASSO coefficient profiles of the target genes with nonzero coefficients determined by the optimal lambda. (E) Distribution of risk score, survival time, and heatmap of four prognostic genes expression in the TCGA cohort. (F) The Kaplan–Meier survival analysis for HCC patients at the high‐ or low‐risk group in TCGA. (G) ROC curves for predicting 1‐, 3‐, 5‐year overall survival in TCGA

Furthermore, the univariate and multivariate Cox regression analyses were conducted to evaluate the target gene as an independent prognostic factor in the TCGA cohort. The univariate Cox analysis demonstrated that all target genes were significantly correlated with clinical prognosis in HCC patients. Among them, *CDC20*, *PTTG1*, and *UBE2S* were high‐risk factors (hazard ratio > 1), and *DNASE1L3* and *SOCS2* were protective factors (hazard ratio < 1) (Figure 7A). However, multivariate Cox regression analysis showed that only *CDC20* and *SOCS2* were independent predictors for HCC prognosis (Figure 7B).

Next, LASSO regression analysis with tenfold cross‐validation was conducted to select the most predictive genes as prognostic indicators. The coefficients for corresponding genes were generated according to the partial likelihood deviance and determined with its lowest value at a log λ = −4.4 (Figure 7C,D). Eventually, four genes (*UBE2S*, *CDC20*, *DNASE1L3*, and *SOCS2*) were enrolled to construct the prognostic signature using the formula: risk score = (0.0465) × *UBE2S* + (0.1851) × *CDC20* + (−0.0461) × *DNASE1L3* + (−0.2279) × *SOCS2*. The patients were further assigned to the high‐ or low‐risk groups using the median risk score as the cut‐off point (Figure 7E). The Kaplan–Meier survival curves revealed a significant difference in overall survival between groups. The high‐risk patients showed a worse prognosis compared with the low‐risk patients (Figure 7F). Moreover, ROC curve analysis demonstrated the predictive ability of the risk score for 1‐, 3‐ and 5‐year overall survival, with areas under the curve (AUCs) of 0.77, 0.723, and 0.706, respectively (Figure 7G).

## 4 DISCUSSION

Liver cancer is a highly heterogeneous disease, and the complex mechanism behind it needs more thorough understanding. To date, the prognostic tools used to assess HCC patient risk remain undesirable.^29^ Advancements in genetic research have allowed more insights into the mechanism behind this malignant disease and may provide more advanced and accurate ways to evaluate the prognosis of HCC patients. Genetic biomarkers have been identified for cancer detection, risk assessment, and prognosis prediction in multiple types of cancer, including brain cancer, colorectal cancer, and prostate cancer.^30^, ^31^, ^32^ Genetic tools can also help in cancer prevention and treatment by providing precision therapeutic targets, which have been proven to be effective in breast cancer treatment.^33^, ^34^ In this study, we identified five target genes (*UBE2S*, *PTTG1*, *CDC20*, *SOCS2*, and *DNASE1L3*) closely correlated with the prognosis of HCC patients through the integration of gene expression profiles from multiple databases. Using these prognostic genes, we eventually constructed a prognostic model for predicting the survival of HCC patients.

Among the five prognostic‐related genes, expression of *UBE2S*, *PTTG1*, and *CDC20* was up‐regulated, whereas *SOCS2* and *DNASE1L3* were down‐regulated in HCC tissues. UBE2S belongs to the ubiquitin‐conjugating enzyme (E2) family and is critical in cell cycle regulation, cell differentiation, and DNA repair.^5^, ^35^ In HCC, UBE2S has been observed to enhance the ubiquitination of p53 for protein degradation in HCC cells.^36^ PTTG1 is a securin protein that inhibits sister–chromatid separation, which is associated with tumorigenesis by promoting cancer cell proliferation, migration, and invasion. PTTG1 also plays an important role in HCC growth and metastasis cascade via activating PI3K/AKT signalling pathway and epithelial–mesenchymal transition‐related factors.^37^ CDC20 activates the anaphase‐promoting complex and, in turn, modulates mitotic exit.^38^, ^39^ It has been shown that CDC20 is vital in HCC cells’ proliferation by mediating PHD3 ubiquitination and HIF‐1α activation.^40^ SOCS2 is a member of the suppressor of the cytokine signalling pathways. It is a transcriptional repressor in multiple proliferation‐related signalling pathways, and its suppression has been observed in lung, breast, and ovarian cancers.^41^, ^42^, ^43^ SOCS2 inhibits HCC metastases via negatively regulating JAK/STAT signalling pathway in HCC cells.^44^ DNASE1L3 is a member of the deoxyribonuclease I family, which encodes proteins that could digest DNA in chromatin. DNASE1L3 is widely down‐regulated in human cancers, and the down‐regulation of DNASE1L3 is associated with poor prognosis in various types of cancers.^45^, ^46^

The present study examined the expression of target genes in different cell types through single‐cell analysis. We found that the aberrant expression of the genes was mainly present in immune cells, which hinted that the target genes might have affected HCC progression by influencing immune cells. Further analysis revealed that the expression level of the genes has a significant correlation with the infiltration levels of multiple types of immune cells, primarily Treg cells, B cells, monocytes, and DCs, indicating that the target genes might have promoted immune cell infiltration and in turn contributing to cancer proliferation and progression, leading to a worse prognosis.

Notably, the prognostic prediction models used to evaluate HCC patient risk in clinical practice remain undesirable.^29^ In this study, we constructed a risk score system to predict the prognosis of HCC patients using the LASSO regression model. This system included four target genes as prognostic parameters (*UBE2S*, *CDC20*, *SOCS2*, and *DNASE1L3*). *UBE2S* and *CDC20* were positively related factors involving in cell mitosis and cell cycle checkpoint pathways. In contrast, *SOCS2* and *DNASE1L3* were negatively related factors, which were associated with cell cycle regulation. Previous studies have pointed out that cell cycle alterations and mitosis signalling pathways are closely associated with cancer progression and affect cancer immune infiltration.^47^, ^48^, ^49^ Recent studies have also suggested that complement cascade may be linked with tumour‐promoting inflammation and cancer immune infiltration.^50^ Thus, the target genes may contribute to the tumorigenesis and progression of HCC through promoting tumour cell proliferation and immune infiltration. Although our findings showed promising results, additional studies are needed to define the underlying molecular mechanisms.

In conclusion, we constructed a promising gene prognostic signature based on multiple databases for predicting clinical outcomes in patients with HCC. This individualized risk score signature could effectively conduct risk stratification, survival prediction, and immune microenvironment evaluation for HCC patients, which would be conducive to clinical decision‐making and personalized treatment.

## CONFLICT OF INTEREST

All authors declare no conflict of interest.

## AUTHOR CONTRIBUTION

**Enjiang Lai:** Data curation (equal); Formal analysis (equal); Investigation (equal); Methodology (equal); Writing‐original draft (equal); Writing‐review & editing (equal). **Yang Tai:** Data curation (equal); Formal analysis (equal); Investigation (equal); Methodology (equal); Validation (equal); Writing‐original draft (equal); Writing‐review & editing (equal). **Jingsun Jiang:** Data curation (equal); Project administration (equal); Resources (equal). **Chong Zhao:** Data curation (equal); Formal analysis (equal); Investigation (equal). **Yang Xiao:** Data curation (equal); Investigation (equal). **Xin Quan:** Investigation (equal); Methodology (equal); Validation (equal). **Hao Wu:** Conceptualization (equal); Data curation (equal); Formal analysis (equal); Funding acquisition (equal). **Jinhang GAO:** Conceptualization (lead); Funding acquisition (lead); Supervision (lead); Writing‐review & editing (lead).

## Supporting information

Fig S1‐S10

## Data Availability

All data are present in the manuscript. All data are available from the corresponding author Jinhang Gao (Gao.jinhang@scu.edu.cn or Gao.jinhang@qq.com) under reasonable request.
