# Supplementary material for: Prognostic evaluation and immune infiltration analysis of five bioinformatic selected genes in hepatocellular carcinoma
Source: J Cell Mol Med. 2021 Nov 2;25(24):11128–41. doi: 10.1111/jcmm.17035 (PMC8650024; doi:10.1111/jcmm.17035)
Supplement: Supplementary file 1 — Fig S1‐S10 [file JCMM-25-11128-s001.pdf]

Supporting information

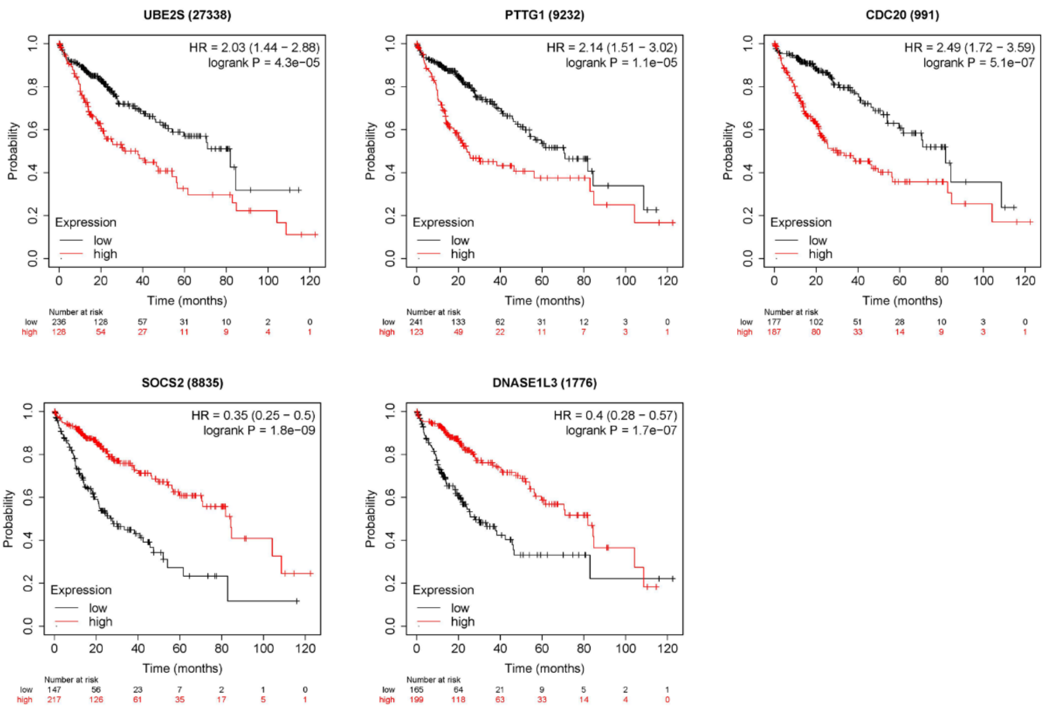

Supporting Figure S1. Prognostic value of the target genes in HCC (Kaplan-Meier Plotter).

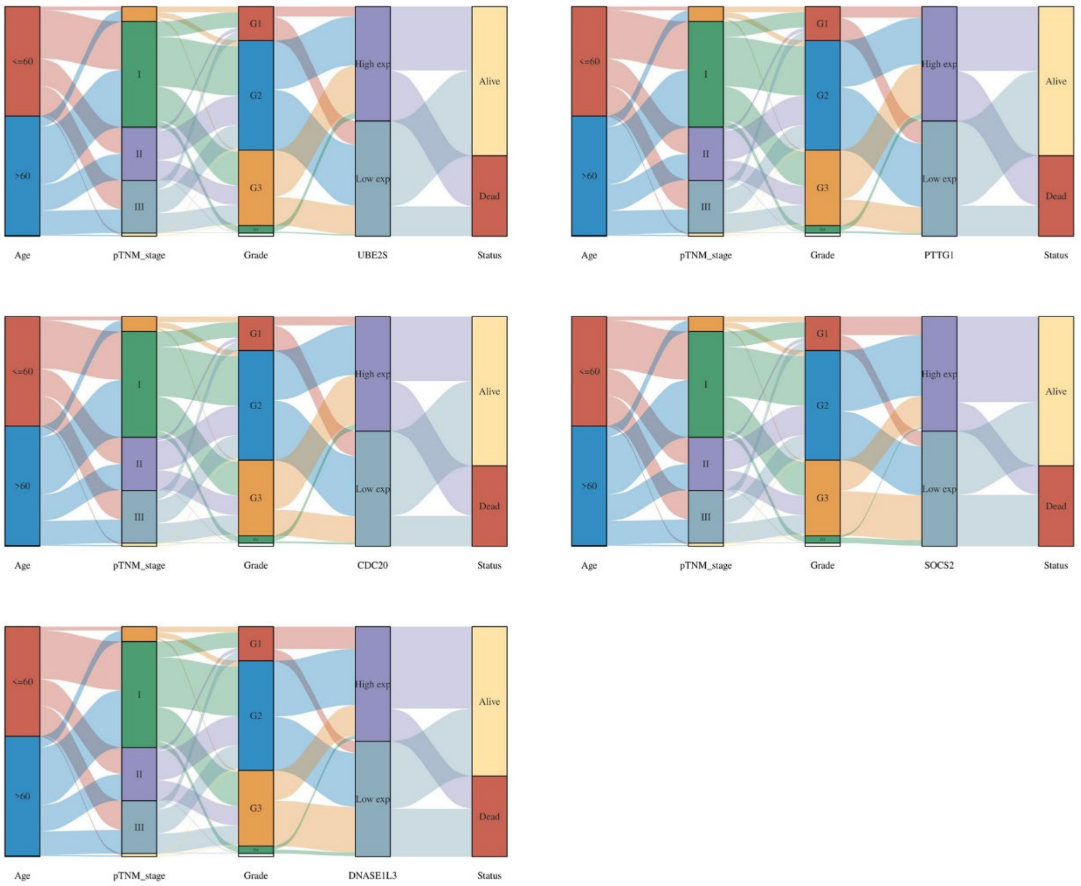

Supporting Figure S2. Sankey diagrams showed the correlation between gene expression, clinicopathological characteristics and prognosis in HCC patients (TCGA).

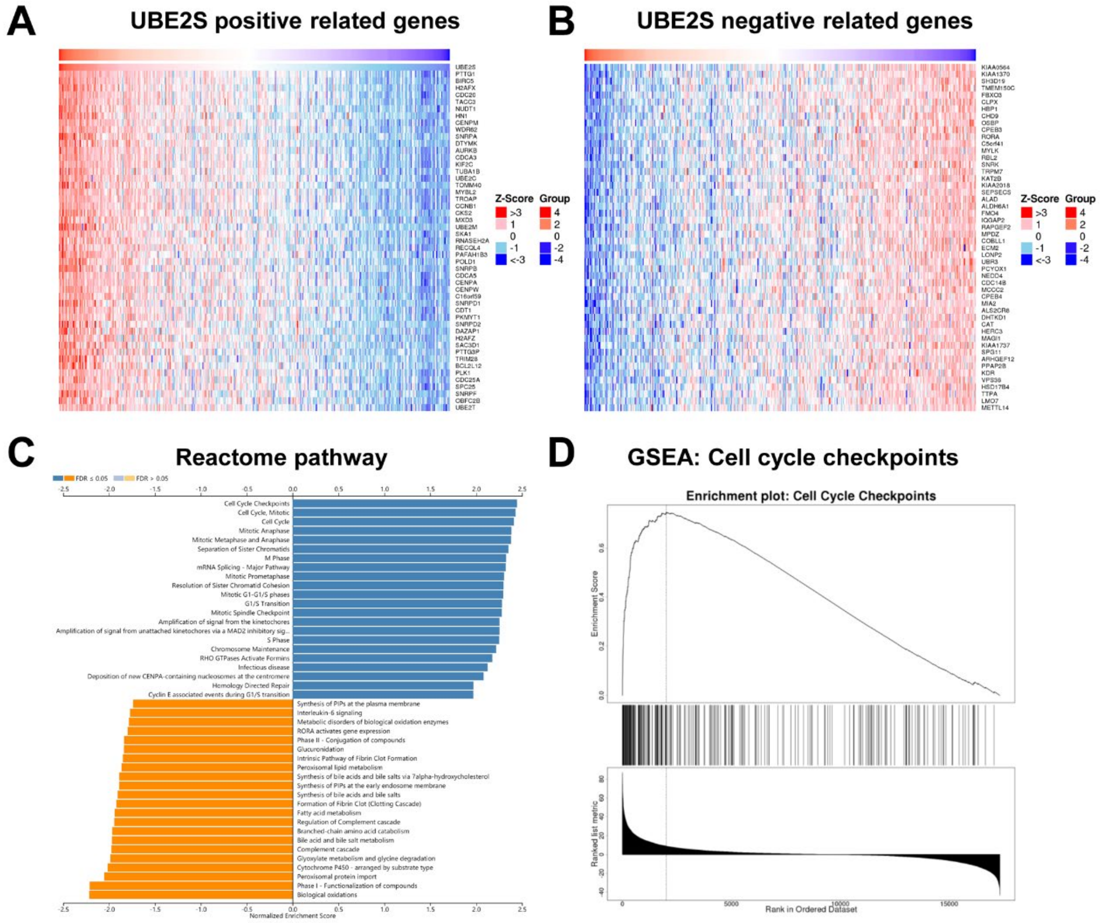

Supporting Figure S3. Gene co-expression analysis and pathway enrichment of *UBE2S* (LinkedOmics).

A: Heatmap of the genes positively correlated with *UBE2S*. **B:** Heatmap of the genes negatively correlated with *UBE2S*. **C:** Reactome pathway enrichment analysis of *UBE2S*-related genes. **D:** GSEA of cell cycle checkpoint signaling pathway target genes. HCC enriched genes were plotted to the left and control enriched genes were plotted to the right.

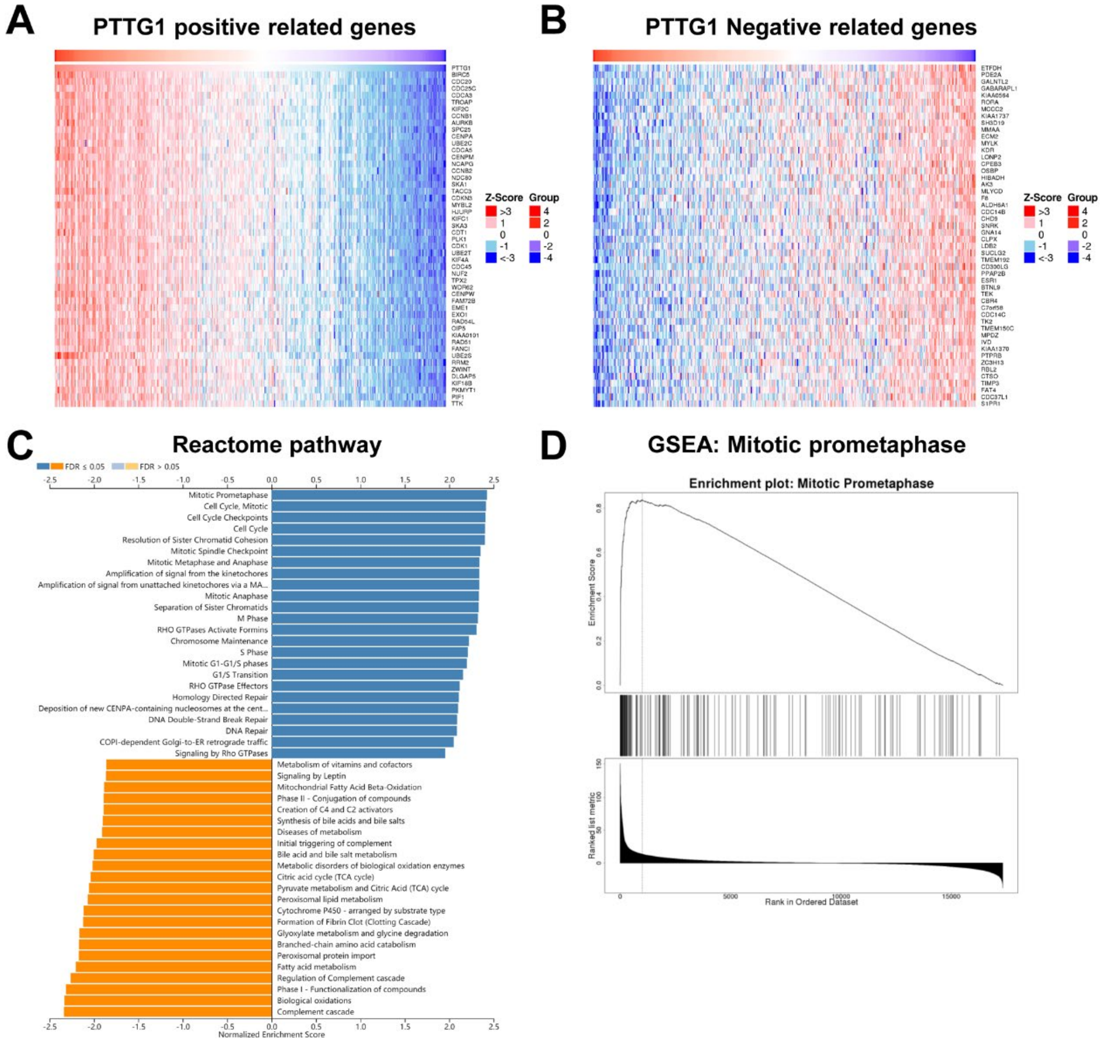

Supporting Figure S4. Gene co-expression analysis and pathway enrichment of *PTTG1* (LinkedOmics).

A: Heatmap of the genes positively correlated with *PTTG1*. **B:** Heatmap of the genes negatively correlated with *PTTG1*. **C:** Reactome pathway enrichment analysis of *PTTG1*-related genes. **D:** GSEA of mitotic prometaphase signaling pathway target genes. HCC enriched genes were plotted to the left and control enriched genes were plotted to the right.

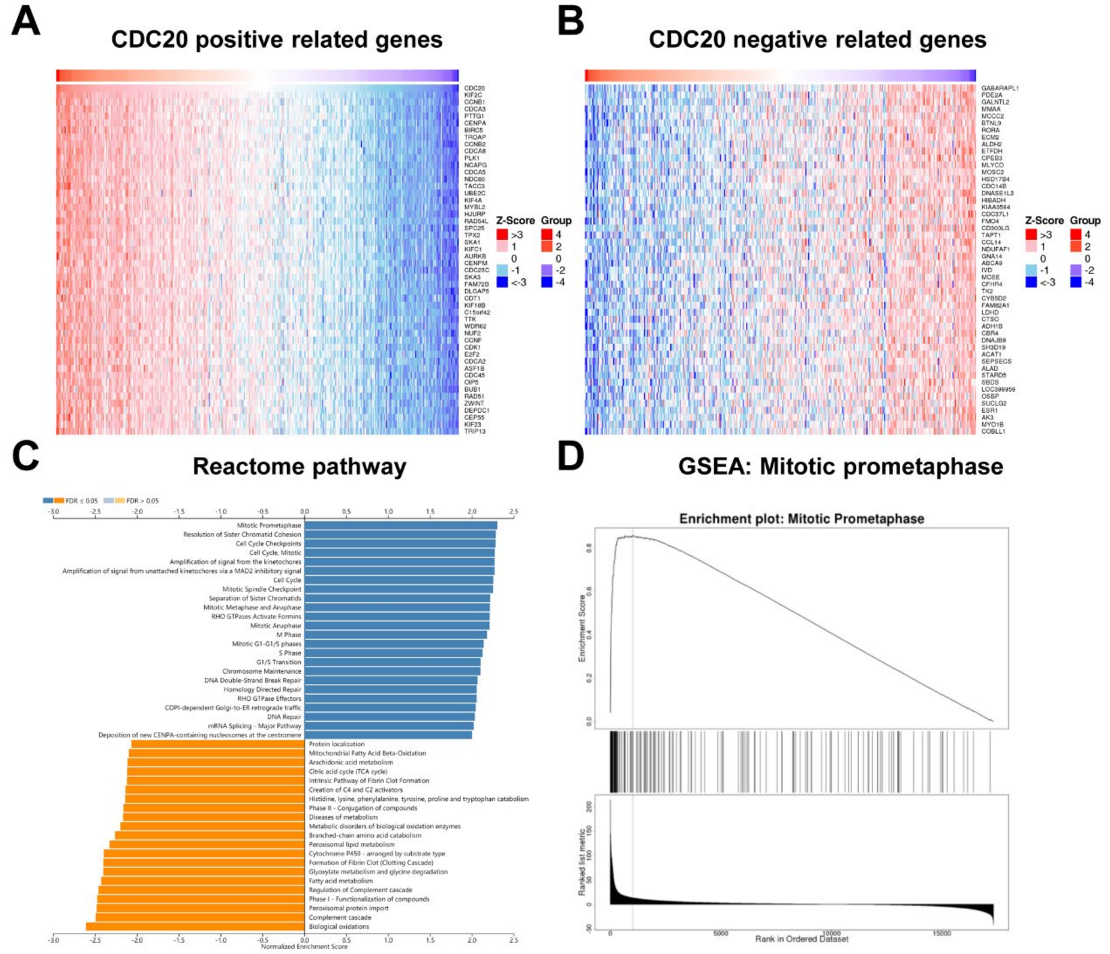

Supporting Figure S5. Gene co-expression analysis and pathway enrichment of *CDC20* (LinkedOmics).

A: Heatmap of the genes positively correlated with *CDC20*. **B:** Heatmap of the genes negatively correlated with *CDC20*. **C:** Reactome pathway enrichment analysis of *CDC20*-related genes. **D:** GSEA of mitotic prometaphase signaling pathway target genes. HCC enriched genes were plotted to the left and control enriched genes were plotted to the right.

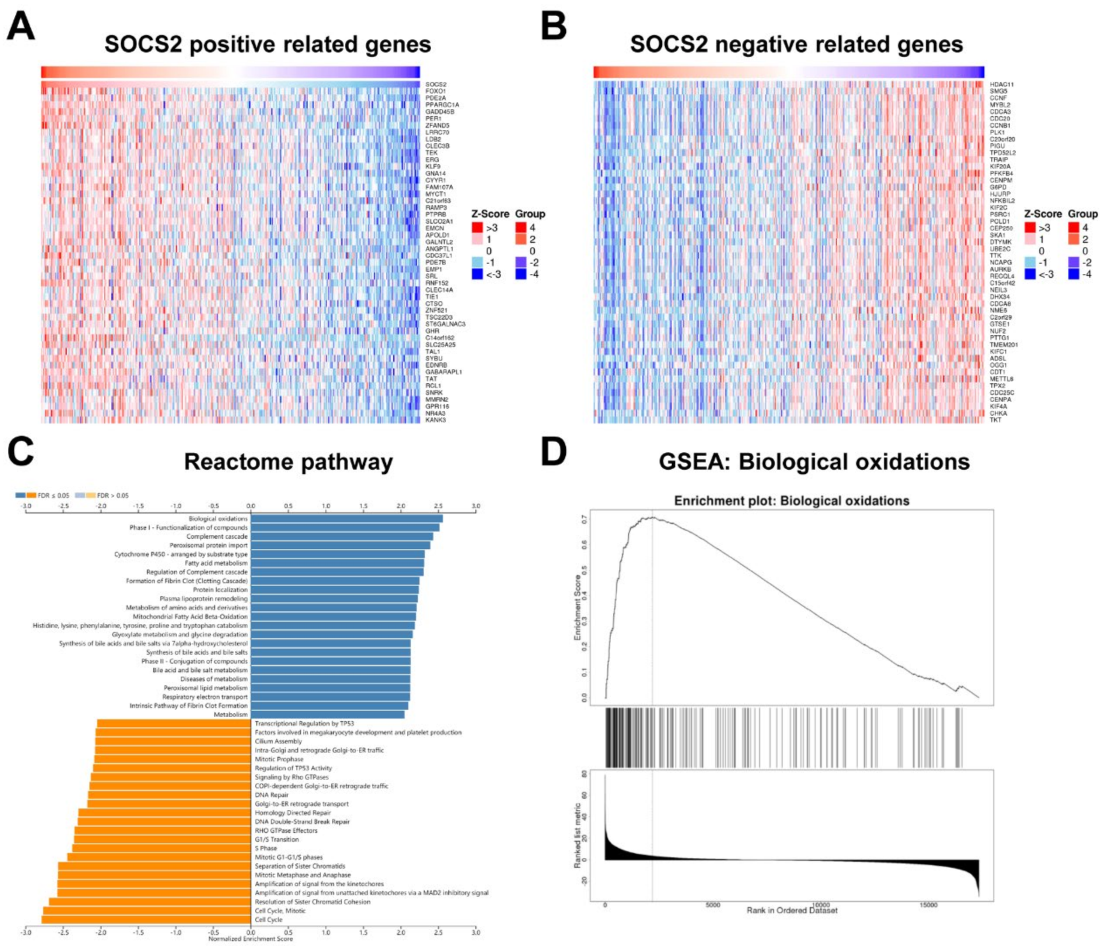

Supporting Figure S6. Gene co-expression analysis and pathway enrichment of *SOCS2* (LinkedOmics).

A: Heatmap of the genes positively correlated with *SOCS2*. **B:** Heatmap of the genes negatively correlated with *SOCS2*. **C:** Reactome pathway enrichment analysis of *SOCS2*-related genes. **D:** GSEA of biological oxidations signaling pathway target genes. HCC enriched genes were plotted to the left and control enriched genes were plotted to the right.

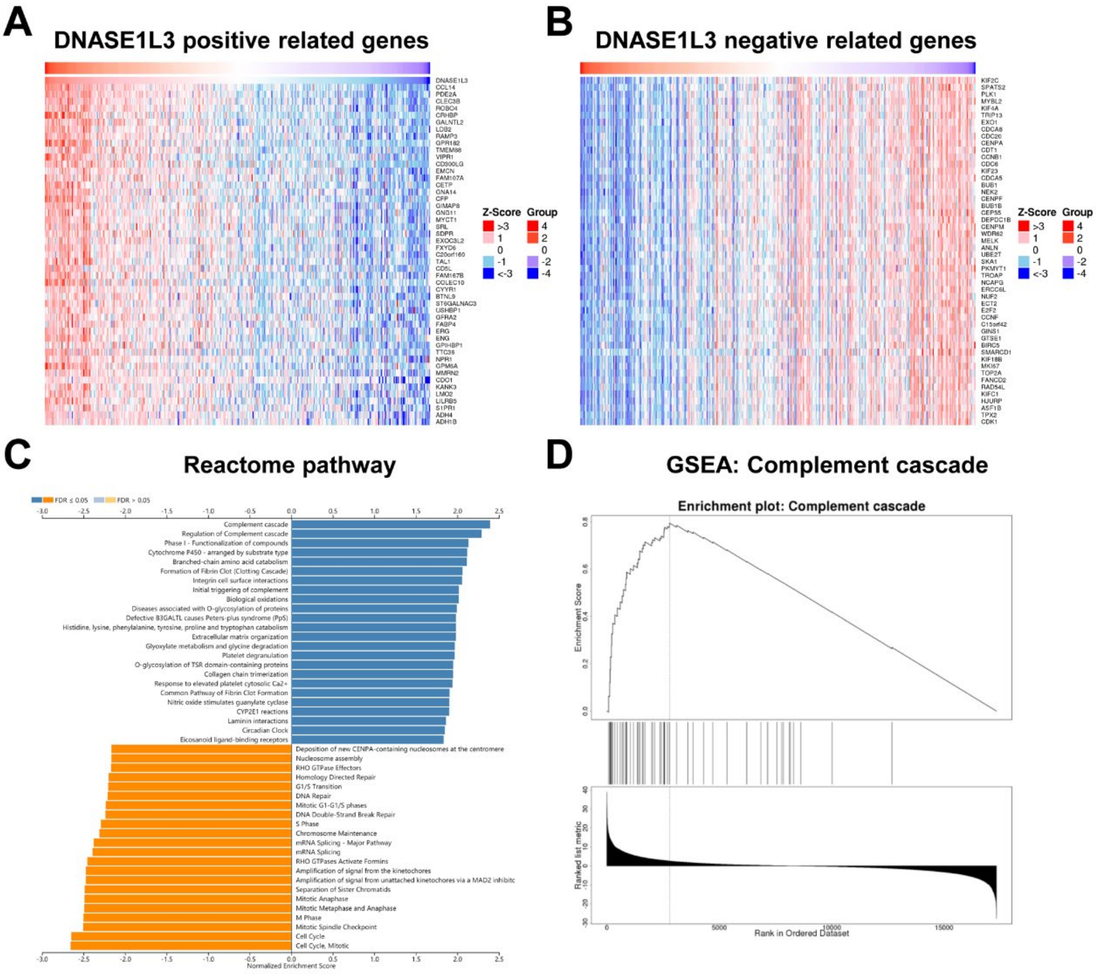

Supporting Figure S7. Gene co-expression analysis and pathway enrichment of *DNASE1L3* (LinkedOmics).

A: Heatmap of the genes positively correlated with *DNASE1L3*. **B:** Heatmap of the genes negatively correlated with *DNASE1L3*. **C:** Reactome pathway enrichment analysis of *DNASE1L3*-related genes. **D:** GSEA of complement cascade signaling pathway target genes. HCC enriched genes were plotted to the left and control enriched genes were plotted to the right.

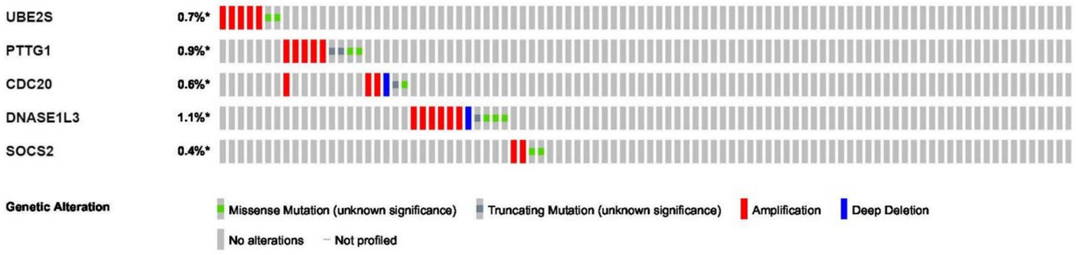

Supporting Figure S8. Mutation analysis of the target genes in HCC (cBioPortal).

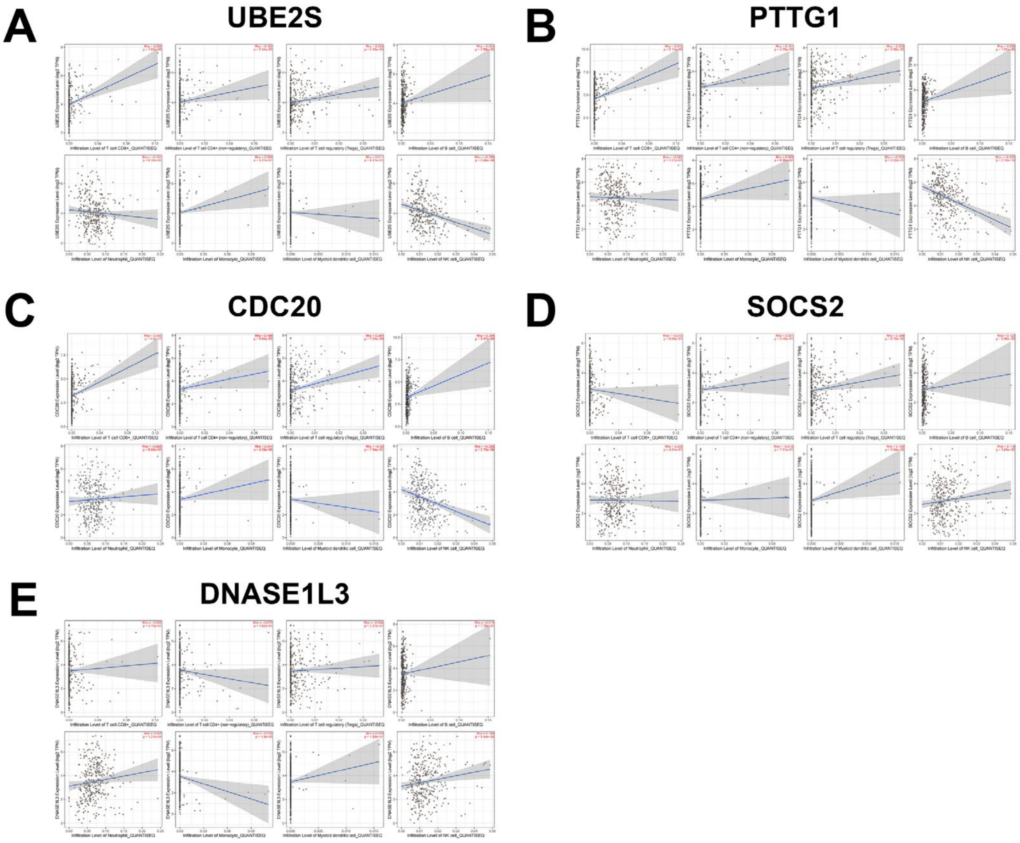

Supporting Figure S9. Correlation between the target genes and immune cells infiltration in HCC.

Partial Spearman's correlation analysis between the expression of *UBE2S* (A), *PTTG1* (B), *CDC20* (C), *SOCS2* (D), and *DNASE1L3* (E) and infiltration of different immune cells ($CD8^+$ T cells, $CD4^+$ T cells, regulatory T cells, B cells, neutrophils, monocytes, myeloid dendritic cells, and natural killer cells) in HCC (TIMER).

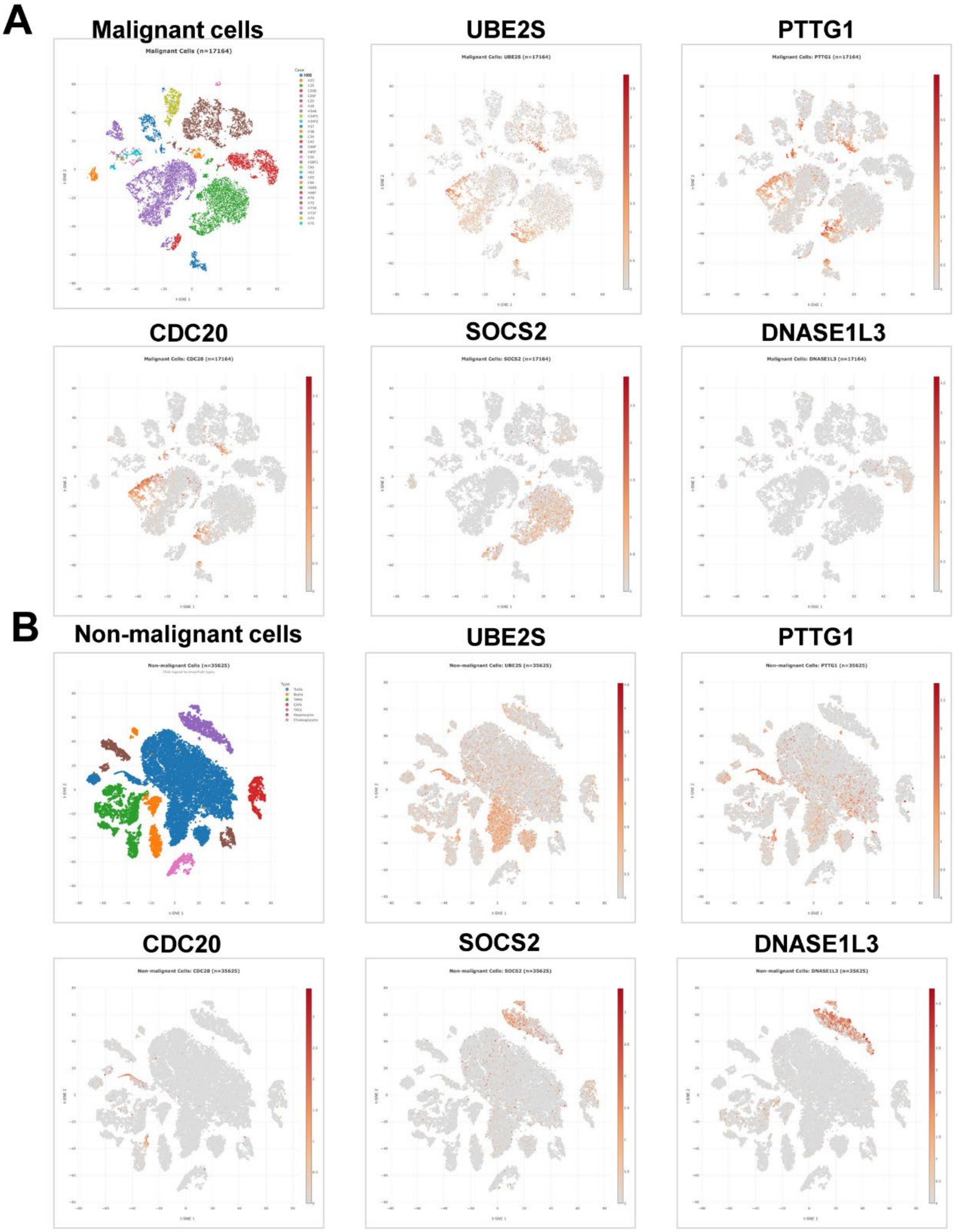

Supporting Figure S10. Single-cell analysis of the target genes in HCC.

t-SNE analysis of the target genes in malignant cells (A) and non-malignant cells (B) in HCC (scAtlasLC).
